# Perspectives of cyanobacterial cell factories

**DOI:** 10.1007/s11120-023-01056-4

**Published:** 2023-11-15

**Authors:** Anastasios Melis, Diego Alberto Hidalgo Martinez, Nico Betterle

**Affiliations:** 1 Department of Plant and Microbial Biology, University of California, MC-3102, Berkeley, CA 94720-3102 USA; 2 Present Address: Department of Biology, Healthcare and the Environment, Faculty of Pharmacy and Food Sciences, University of Barcelona, Barcelona, Spain; 3 Present Address: SoLELab, Department of Biotechnology, University of Verona, 37134 Verona, Italy

**Keywords:** Biopharmaceutical proteins, Fusion constructs, Phycocyanin, Plant essential oils, Protein overexpression, *Synechocystis* sp. PCC 6803

## Abstract

Cyanobacteria are prokaryotic photosynthetic microorganisms that can generate, in addition to biomass, useful chemicals and proteins/enzymes, essentially from sunlight, carbon dioxide, and water. Selected aspects of cyanobacterial production (isoprenoids and high-value proteins) and scale-up methods suitable for product generation and downstream processing are addressed in this review. The work focuses on the challenge and promise of specialty chemicals and proteins production, with isoprenoid products and biopharma proteins as study cases, and the challenges encountered in the expression of recombinant proteins/enzymes, which underline the essence of synthetic biology with these microorganisms. Progress and the current state-of-the-art in these targeted topics are emphasized.

## Preface – why cyanobacteria?

Cyanobacteria, also known as blue–green algae, are a group of photosynthetic microorganisms that can use the energy of sunlight to convert carbon dioxide (CO_2_) and water (H_2_O) into valuable products (Jansson 2012; Berla et al. 2013; Savakis and Angermayr 2013; Santos-Merino et al. 2019). Advantages afforded by cyanobacteria include the extensive genomic information from a number of species, ease and precise manipulation of cyanobacterial genomes, including double homologous recombination to enable insertion of transgenes or multigene operons and antibiotic selectable markers, or deletion of specific endogenous genes from the cellular DNA (Satta et al. 2023). Important practical features of these microorganisms include the much faster growth rate relative to plants (Melis 2009) and the ability to avoid contaminants in mass liquid cultures (Chaves et al. 2015; Xie et al. 2017).

Significant advances in cyanobacterial cell factories were recently realized, including genetically engineered strains to perform more efficiently under bright sunlight and high biomass density, strains able to use high concentrations of CO_2_ to support high rates of photosynthesis, and also be modified to direct their metabolism toward the production of specific high-value proteins and chemicals (Bentley and Melis 2012; Kirst et al. 2014; Yu et al. 2015, Jaiswal et al. 2018; Betterle et al. 2020; Włodarczyk et al. 2020). Progress in this field has also enabled the development of tools for a more efficient and cost-effective engineering of these microorganisms for specific biotechnological applications (Albers et al. 2015; Santos-Merino et al. 2019), although the development of such tools in cyanobacteria lags behind those in conventional heterotrophic microorganisms (Cheng et al. 2023).

Recently, CRISPR-based tools have gained attention in cyanobacterial research for their ability to target polyploid genomes in these organisms and to increase the efficiency of cyanobacterial transformation (Behler et al. 2018; Sengupta et al. 2022; Patel et al. 2023). Moreover, a further advancement of CRISPR, CRISPRi, which was based on the use of a deactivated Cas enzyme, was described as an efficient method for functional studies based on gene silencing (Gordon et al. 2016; Yao et al. 2016; Liu et al. 2020).

A major area of progress has been the optimization of cyanobacterial growth capacity. This includes identification of the genetic determinants required for the rapid growth of cyanobacterial strains (Yu et al. 2015; Mills et al. 2022), as well as for enhancing their natural transformation ability (Wendt et al. 2022). These efforts have resulted in faster growth rates and higher biomass yields, which can translate into greater yields of heterologous product synthesis and lower production costs.

**Fig. 1:**
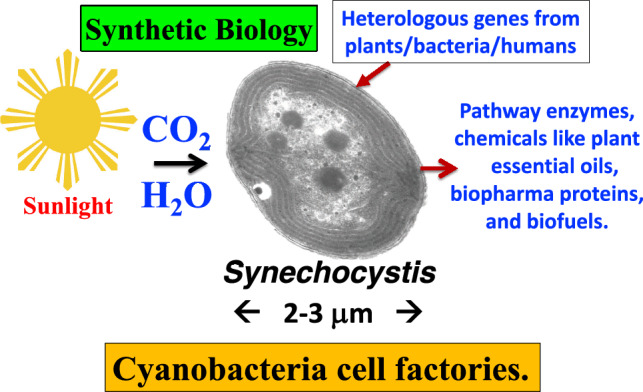
Schematic of the cyanobacterial cell factories platform with *Synechocystis* as the model organism. Sunlight, CO_2_, and H_2_O are inputs to the process whereas heterologous pathway enzymes, and chemicals like plant essential oils, biofuels, and recombinant biopharma proteins are target outputs

While much of the work on cyanobacterial cell factories has been conducted in the laboratory, there is now interest in scaling up production to pilot or industrial levels (Novoveská et al. 2023). Such effort would require optimization of the cultivation conditions, development of efficient downstream processing, and innovative product isolation approaches (Pandey et al. 2022). Nevertheless, the above-mentioned advances in cyanobacterial synthetic biology have enabled the engineering of modified strains that can direct metabolism toward a variety of products with commercial significance. Metabolically engineered cyanobacteria have successfully produced compounds such as ethanol, butanol, lactic acid, ethylene, terpene hydrocarbons, and other interesting products (Satta et al. 2023). Other efforts have included synthesis and accumulation of biopharmaceutical proteins, isoprenoid pathway enzymes, chemicals like plant essential oils, and potentially biofuels (Fig. 1).

Overall, advances in cyanobacterial cell factories are opening up new possibilities for sustainable production of a variety of valuable compounds. Continued R&D in this field is likely to lead to further breakthroughs in years to come that could have a significant impact on this technology and its applications in the bioeconomy.

### Isoprenoid products

**Fig. 2:**
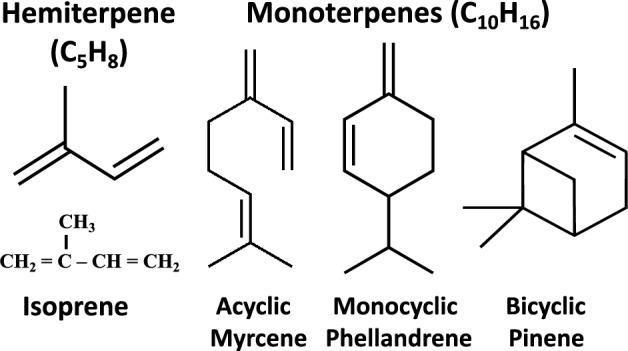
The chemical formula of the hemiterpene isoprene (C_5_H_8_), the acyclic β-myrcene, the monocyclic β-phellandrene, and the bicyclic β-pinene monoterpene (C_10_H_16_) hydrocarbons

The simplest terpene hydrocarbons are the hemiterpene isoprene (2-methyl-1,3-butadiene; C_5_H_8_) and monoterpenes (C_10_H_16_), as exemplified by the acyclic myrcene, monocyclic phellandrene, and bicyclic pinene (Fig. 2). However, there are many other monoterpenes, identified from a variety of plants, as essential oils and fragrance chemicals, including camphene, sabinene, δ-3-carene, α-terpinene, *p*-cymene, limonene, β-ocimene, γ-terpinene, and terpinolene (Gomes et al. 2013), among others. Whereas, isoprene and monoterpenes have received the majority of attention in the cyanobacterial literature (Pattanaik and Lindberg 2015; Kant et al. 2023), higher-order sesquiterpenes (C_15_H_24_), diterpenes (C_20_H_32_), and triterpenes (C_30_H_50_) have also been investigated as putative biotechnology products of cyanobacterial cell factories (Rautela and Kumar 2022). Moreover, oxygenated / hydroxylated terpenes, e.g., patchoulol (C_15_H_25_OH; Dienst et al. 2020) and geranyllinalool (C_20_H_33_OH; Formighieri and Melis 2017), have been the subject of research and development, as possible cell factory products. Lastly, renewable carotenoids production has also been of interest in the field (Liu et al. 2019; Menin et al. 2019; Diao et al. 2020).

#### Isoprene

Isoprene is a volatile 5-carbon organic compound that is naturally generated by a variety of organisms, notably terrestrial herbaceous and deciduous plants (Loreto and Sharkey 1990; Sharkey et al. 2005, 2007; Guenther et al. 2006; Harrison et al. 2013), some types of bacteria (Kuzma and Fall 1993; Fall and Copley 2000; Bäck et al. 2010), and even fungi and humans (Mochalski et al. 2023). Of interest is the case of terrestrial plants, in which expression of the endogenous isoprene synthase gene is induced upon heat stress, resulting in substantial amounts of photosynthetic substrate conversion to isoprene (Sharkey et al. 2007). The latter readily diffuses through the chloroplasts, cell membranes, and walls and exits the leaves through the stomata, acting as a thermotolerance mechanism for the plant. The thermotolerance interpretation is based on the observation that isoprene-emitting leaves are able to quickly recover after heat stress, suggesting a mitigation of heat damage (Velikova and Loreto 2005).

A growing interest in using cyanobacteria as a heterologous source of isoprene has followed the pioneering work of Lindberg et al. (2010). Conferring heterologous isoprene production to cyanobacteria is complex and involves multiple steps. First, the genes responsible for isoprene synthesis were identified, cloned, codon-optimized, and genetically installed into the cyanobacterial genome (Lindberg et al. 2010; Bentley and Melis 2012; Bentley et al. 2014). The expression of these genes, at the protein level, was verified and maximized (Chaves et al. 2017) in order to enhance the yield of isoprene production, without negatively affecting rates of cellular photosynthesis and cell fitness. Moreover, altering the methylerythritol 4-phosphate (MEP) pathway and, specifically, the dimethylallyl diphosphate (DMAPP) to isopentenyl diphosphate (IPP) ratio toward more DMAPP and less IPP has helped to increase the yield of isoprene, as it increased the substrate (DMAPP) from which isoprene is generated (Chaves et al. 2016; Gao et al. 2016; Chaves and Melis 2018a).

Interest in cyanobacterial isoprene is based on the potential for constitutive production by these photosynthetic microorganisms (Englund et al. 2018; Chaves and Melis 2018b; Ko et al. 2019; Sethia et al. 2019; Zhou et al. 2021; Yadav et al. 2023; Yahya et al. 2023) and the potential to provide a sustainable and renewable source of this important compound for use in a variety of applications. These include the production of synthetic rubber, plastics, adhesives, pesticides, medicines, fragrances, and biofuel derivatives. In this respect, isoprene is in high demand in the commercial sector, with more than one million tonnes, currently produced from fossil fuel resources and commercially consumed annually (Whited et al. 2010; Morais et al. 2015).

With current technology, yield of isoprene production in cyanobacterial cell factories has reached 12.5 mg per g biomass (Chaves and Melis 2018a,b). Flux through the native methylerythritol phosphate isoprenoid pathway naturally consumes about 50 mg of photosynthetically generated metabolites per g biomass produced, which is needed to satisfy the endogenous isoprenoid needs of the cell (Lindberg et al. 2010; Melis 2012; 2013). Hence, the above-mentioned currently achieved isoprene yield of 12.5 mg per g biomass suggested that the installed heterologous pathway to isoprene constitutively achieved flux equal to about 25% of that in the native isoprenoid pathway. Heterologous carbon flux to isoprene was in addition to that consumed for the synthesis of native isoprenoids, based on the observation that isoprene production did not impede cell growth.

However, flux through the isoprenoid biosynthetic pathway need not be limited to the above-mentioned yields. There are examples in the literature, e.g., the green colonial microalga *Botryococcus braunii* variety Showa, whereby carbon partitioning through the isoprenoid biosynthetic pathway consumes ~ 45% of the cellular endogenous metabolites, most of which is invested in the synthesis and extracellular accumulation of the biologically aberrant triterpenoid botryococcene (Melis 2013). Thus, it is theoretically possible to enhance the carbon partitioning and flux of the cyanobacterial isoprenoid biosynthetic pathway to achieve far greater than present product yields.

#### Monoterpenes, higher-order Isoprenoids, and other cyanobacterial bio-products

Cyanobacterial limonene (Davies et al. 2014; Jongedijk et al. 2016; Lin et al. 2021; Li et al. 2022) and β-phellandrene production (Bentley et al. 2013; Formighieri and Melis 2015; 2016; Xie et al. 2017; Lin and Pakrasi 2019; Betterle and Melis 2019) have attracted attention in the field. Production of sesquiterpenes (C_15_H_24_) like farnesene (Lee et al. 2017), bisabolene (Davies et al. 2014), and caryophyllene (Reinsvold et al. 2011) have also been reported.

Several reviews on the cyanobacterial production of other useful chemicals (Ducat et al. 2011; Machado and Atsumi 2012; Ungerer et al. 2012; Savakis and Hellingwerf 2015; Pattanaik and Lindberg 2015; Davies et al. 2015; Woo 2017; Wang et al. 2020; Rodrigues and Lindberg 2021b; Rautela and Kumar 2022; Barone et al. 2023) amplify the breadth and depth of this growing field. These will not be discussed further in the present “Perspectives.” The interested reader is referred to the relevant review articles cited in this section.

### Spontaneous product separation from the biomass and the liquid culture

Critical issues in the field of isoprenoids production include the yield of the process. Inherent limitations including (i) flux of endogenous metabolites, comprising the products of photosynthesis, to the isoprenoid biosynthetic pathway (Melis 2013) and (ii) product separation from the biomass and the liquid culture. As discussed above, flux of endogenous metabolites through the MEP pathway for the synthesis of all cellular isoprenoids is typically limited to about 5% of total cellular carbon (Lindberg et al. 2010). Efforts to enhance this highly regulated flux in cyanobacteria included installation of the heterologous mevalonic acid pathway in isoprene and β-phellandrene competent *Synechocystis* sp. PCC 6803 (hereafter referred to as *Synechocystis)*, a property that substantially enhanced the yield of isoprene (Bentley et al. 2014) and β-phellandrene accumulation (Formighieri and Melis 2015; 2016; Betterle and Melis 2019).

The interplay between biomass accumulation and heterologous product generation is also of interest and pertinent to cell factories. In theory, biomass accumulation and heterologous product generation (biofuels or chemicals) are assumed to be competing processes (Melis 2012). In this respect, it is intriguing that preliminary observations have shown enhancement in the rate of light-saturated photosynthesis, when a heterologous process adds a new metabolic sink, e.g., when isoprene or β-phellandrene biosynthetic pathways are installed, without a negative effect on the rate of biomass accumulation (Bentley et al. 2013). This was interpreted to mean that the rate of photosynthesis under saturating illumination is sink limited by the rate of biomass accumulation, rather than by the rate of the light reactions and that photosynthesis can be enhanced when additional carbon-sinks are installed. Thus, a heterologous sink that is separate and apart from the cellular biomass may hold promise in terms of enhancing the light-saturated rate of photosynthesis.

Spontaneous product diffusion (efflux) from the cells and exclusion from the liquid phase of the culture confers a substantial advantage with respect to downstream processing, as it simplifies product isolation and lowers the cost of production. Moreover, it alleviates product inhibition of cellular endogenous metabolism and mitigates potentially adverse effects on cell vitality and growth. In general, product separation from the cells and from the liquid culture proved to be necessary for continuous substrate flux through the heterologous pathway, ensuring product accumulation, while preserving cell fitness. Heterologous products trapped inside the transformant cells are typically detrimental to cell fitness and tend to inhibit cell growth and productivity. Examples of the latter in cyanobacteria include efforts to make botryococcene, a C_30_H_50_ a biologically aberrant triterpene analogous to squalene, and cannabidiol, or CBD oil, a C_21_H_30_O_2_ hybrid of the fatty acid and isoprenoid biosynthetic pathways. Both of these heterologous products proved to be too large for spontaneous diffusion and exclusion from the cells, resulting in cell growth inhibition and loss of productivity (unpublished results). Low amounts of the natural triterpene squalene (C_30_H_50_), however, accumulated in transformant *Synechocystis*, showing that squalene was tolerated and was not detrimental to growth, in spite of the intracellular sequestration of this compound (Englund et al. 2014; Choi et al. 2017; Pattanaik et al. 2020).

Of interest is the case of geranyllinalool, a C_20_H_33_OH diterpenol useful as a fragrance and a feedstock for the synthesis of the antimalarial drug “teprenone.” Geranyllinalool in *Synechocystis* displayed limited spontaneous diffusion from the cells (Formighieri and Melis 2017). It was primarily sequestered inside the transformant cells, corresponding to 60–70% of the total heterologous product, instead of being entirely exuded, as the case is with shorter heterologous terpene hydrocarbons. Extraction of geranyllinalool necessitated disruption of the cells in order to enable release and quantitative isolation of this chemical product. Moreover, geranyllinalool accumulation in the cells caused a mild inhibitory effect on cell fitness and a decline in biomass growth rate such that the duplication time of geranyllinalool-producing *Synechocystis* transformants was 1.4-fold longer than that of the control. The remaining 30–40% of the geranyllinalool product, which diffused out of the cells, was found to float on the surface of sealed liquid cultures (Formighieri and Melis 2017).

Of interest is also the application of dodecane (5% (v/v)) as a non-miscible organic layer on the surface of liquid cultures, designed to help extract from cells and contain medium- and larger-size isoprenoids, such as limonene and bisabolene (Davies et al. 2014; Lee et al. 2017; Rodrigues and Lindberg 2021a). Product titers were reportedly higher, when a dodecane overlay was applied during culturing, suggesting either that dodecane traps large quantities of volatile limonene and α-bisabolene that would otherwise be lost to evaporation and/or that continuous product removal in dodecane alleviates product feedback inhibition, thus promoting higher rates of synthesis.

In summary, spontaneous or assisted (via dodecane) product diffusion out of the cells and exclusion from the liquid phase of the culture affords a substantial advantage in commercialization as (i) it alleviates product inhibition of cellular endogenous metabolism and (ii) it lowers the cost of downstream processing, as it helps to avoid the need for culture dewatering, cell fractionation, and product extraction from the cells.

### Provision of carbon dioxide (CO_2_)

**Fig. 3:**
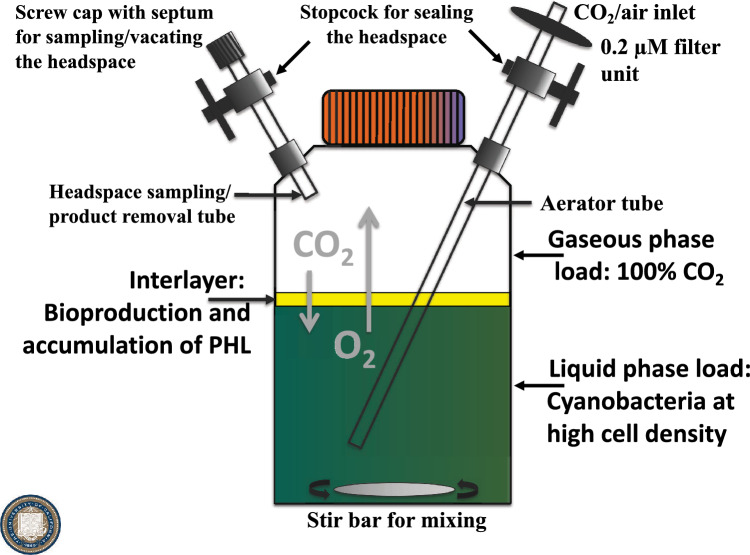
The “Bentley bottle,” a lab-designed 1-L sealed bioreactor for diffusion-based delivery of CO_2_ and O_2_ gas exchange occurring concomitantly with terpene hydrocarbons production and release/accumulation. A 100% CO_2_ gas stream was slowly bubbled into the gaseous/aqueous two-phase medium, calibrated to flush the liquid phase and fill the headspace prior to sealing the reactor. Efficient and spontaneous uptake and assimilation of headspace CO_2_ by the cells occurred in the fully sealed reactor by diffusion and was concomitantly replaced by photosynthetically produced O_2_ and hydrophobic isoprenoids during cell photoautotrophic growth. The latter (O_2_ and hydrophobic isoprenoids) accumulated in the reactor headspace. This device was successfully tested with isoprene, β-phellandrene, and geranyllinalool, as the target isoprenoid products. Schematic adapted from Bentley and Melis (2012)

**Fig. 4:**
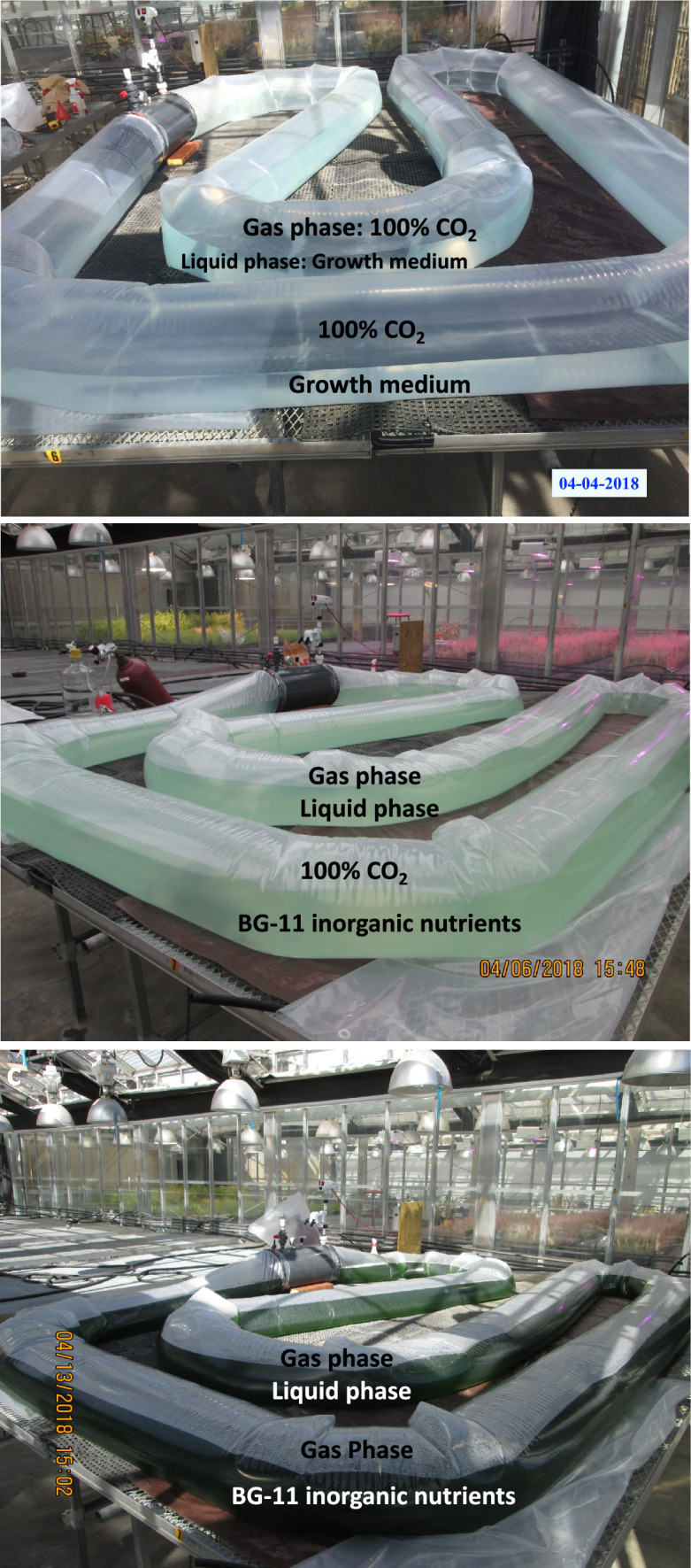
A 1,200-L tubular modular pilot photobioreactor layout in the greenhouse was designed to support cell growth and product generation / accumulation. A control box enabled culture manipulations, including addition of nutrients and removal of culture samples from the reactor. **a** Shown is the gaseous/aqueous two-phase configuration with growth media present but prior to cyanobacterial inoculation. This set-up employed an approximately 50:50 partition between the aqueous and gaseous phases. The latter was filled with a stream of 100% CO_2_. However, the gaseous/aqueous partition ratio could vary depending on organism and growth conditions. **b** View of the reactor described above, immediately following inoculation with a starter culture. In a sealed reactor, this configuration permitted spontaneous diffusion-driven CO_2_ uptake from the gaseous phase and its replacement by O_2_ and by the isoprenoid products generated from the photosynthesis of the cells in the aqueous phase. **c** View of the reactor seven days after inoculation. With ambient sunlight and sufficient CO_2_ substrate, cells grew quickly, resulting in a high-density biomass in the reactor, as evidenced from the high optical density of the culture. Abundance of CO_2_ (100% provision) and bright sunlight helped the rate of cyanobacterial growth and productivity

In addition to cultivation conditions, such as growth temperature and nutrient availability (Rodrigues et al. 2023), provision of CO_2_ (Bentley and Melis 2012) and sunlight utilization efficiency (Melis 2009; Kirst et al. 2014; Hu et al. 2023) are pivotal in efforts to maximize productivity of photosynthetic microorganisms. Bubbling cultures with air, which contains a mere 0.04% CO_2_, poses a stark limitation to the capacity of photosynthesis for cell growth and productivity. This limitation can be alleviated via the use of closed and sealed photobioreactors or other closed cultivation systems, designed specifically for the provision and containment of high amounts of carbon dioxide [100% gaseous CO_2_] to the culture, while at the same time affording containment of volatile isoprenoid products. Of interest in this respect is the design and use of “the Bentley bottle” (Fig. 3, Bentley and Melis 2012), comprising a custom-made fed-batch bioreactor for diffusion-based CO_2_/O_2_ gas exchange and volatile hydrocarbons production and containment. A gas stream comprising 100% CO_2_ was slowly fed into this gaseous/aqueous two-phase bioreactor to flush the aqueous phase and to fill the reactor headspace, upon which the vessel was sealed. Efficient and spontaneous uptake by diffusion and assimilation of headspace CO_2_ by the cells occurred concomitantly with the exchange of photosynthetically produced O_2_ and isoprene or β-phellandrene accumulation during photoautotrophic growth. Accumulation of isoprenoids took place in the sealed bioreactor headspace (Bentley and Melis 2012). The principle of the gaseous/aqueous two-phase bioreactor was successfully tested in an expanded 1200-L pilot bioreactor under ambient conditions in the greenhouse, where the beginning composition of the gaseous phase comprised 100% CO_2_ (Fig. 4a). An illustration of productivity under these conditions was provided upon culture inoculation on 2018-04-06 (Fig. 4b) and the attainment of high cell density in the photobioreactor on 2018-04-13 (Fig. 4c), which showed that growth of cyanobacteria can be robust in the presence of sufficient amounts of CO_2_ and bright sunlight.

### Sunlight utilization efficiency

Cyanobacterial culture productivities can also be improved upon an increase in sunlight utilization efficiency. Cyanobacterial cell factories are converting the energy of sunlight in photosynthesis and storing it as biomass, biofuel, and target chemical or protein/enzyme of interest. The mechanics of this operation require, in addition to non-limiting amounts of CO_2_, ability to absorb all incident sunlight and also ability to convert it in the form of useful photochemistry. The former is satisfied by a sufficiently high density of biomass to ensure absorption of all incident irradiance, which is easily attained. The latter, however, is severely compromised because photosynthetic organisms assemble large arrays of light-absorbing antenna molecules, leading to excessive absorption of sunlight at the surface of the culture, frequently way beyond what they can use biochemically, resulting in a wasteful dissipation of excitation and in a lower than expected culture photochemical productivity. In consequence, cells deeper in such cultures are shaded and do not perform to capacity. A genetic tendency of photosynthetic organisms to assemble large arrays of light-absorbing antenna molecules in their photosystems is a survival strategy and a competitive advantage in the wild (Melis 2009), where sunlight is often limiting (Kirk 1994). Maximum competition of photosynthetic organisms in the wild requires capturing more light for self, even if wasted, and preventing light capture by competing neighbors (Melis 2009). Obviously, this property is detrimental to the yield and productivity in a dense monoculture.

**Fig. 5:**
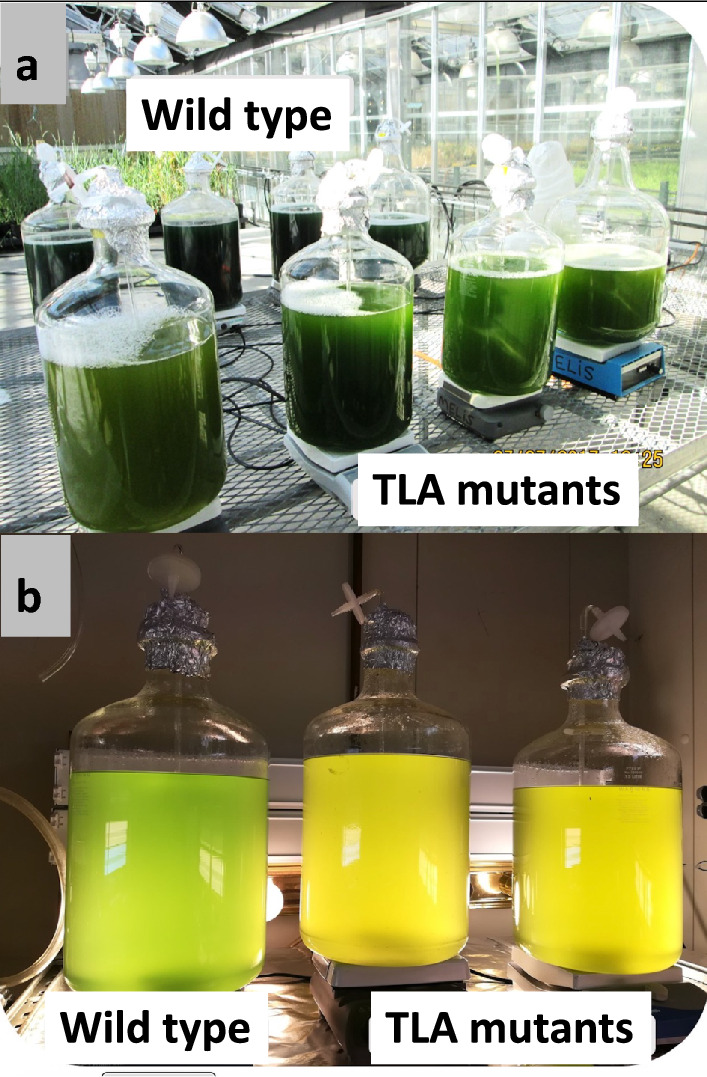
Comparative growth and biomass accumulation of wild-type and **T**runcated **L**ight-harvesting **A**ntenna (TLA) strains of *Synechocystis* 6803 lacking phycocyanin. **a** 13-L carboys with a 12-inch (30 cm) diameter were inoculated with *Synechocystis* wild-type and a phycocyanin-deficient (TLA) mutant, followed by growth in BG-11 inorganic nutrients under ambient conditions in the greenhouse. **b** Early stages of growth for the wild-type and two TLA strains of *Synechocystis* under artificial illumination in the lab

Minimizing, or truncating, the light-harvesting antennae of photosynthesis to maximize culture productivity, i.e., application of the so-called “Truncated Light-harvesting Antenna” (TLA) concept, successfully increased solar energy conversion efficiencies of photosynthesis in tobacco (Kirst et al. 2017; 2018), green microalgae (Polle et al. 2003), and cyanobacteria (Kirst et al. 2014). Generation of TLA strains in all classes of photosynthetic organisms helps to alleviate excess absorption of bright sunlight and the ensuing wasteful dissipation of excitation energy and to maximize solar-to-product energy conversion efficiency and photosynthetic productivity in high-density mass cultivations (Melis 2009). The TLA concept may find application in the commercial exploitation of cyanobacteria for the generation of biomass, biofuels, and chemical feedstocks, as well as nutraceuticals and pharmaceutical proteins. This concept was validated in a side-by-side greenhouse cultivation of *Synechocystis* wild-type and a phycocyanin-deficient (TLA) strain, grown in 13-L volume, 12-inch-diameter carboys in the greenhouse (Fig. 5a). Rates of cell growth for the TLA strains (0.058 g L^−1^ d^−1^) were faster than those for the WT (0.035 g L^−1^ d^−1^) by about 66%, consistent with the lab findings by Kirst et al. (2014). In spite of the greater biomass accumulating in the TLA carboys, cultures in the latter can be seen to having a lighter coloration with an apparent greater transmittance of sunlight through them, as compared to the dark blue–green coloration of the wild type (Fig. 5a). Such differences in culture coloration could also be discerned in early-stage growth in the lab (Fig. 5b). These results with TLA cyanobacteria corroborate similar findings with TLA *Dunaliella salina* (Melis et al. 1999) in which cells with a smaller than wild-type chlorophyll antenna sizes exhibited higher photosynthetic productivities and photon use efficiencies than normally pigmented cells, while displaying greater transmittance of light through a high-density culture.

### Biopharmaceutical and other recombinant protein synthesis

The success of synthetic biology approaches with cyanobacteria and other systems requires overexpression of pathway enzymes and proteins of interest in order to attain higher yields and lower costs. For example, yield of the constitutive generation of isoprenoid compounds in cyanobacteria strongly depends on the concentration of the pathway-catalyzing recombinant enzymes (Chaves et al. 2017; Betterle and Melis 2018; Lim et al. 2020). However, heterologous proteins are almost always not well tolerated by the recipient cell and either pellet as inclusion bodies or are degraded by the cellular proteasome. This is a barrier to the useful application of cyanobacteria in synthetic biology, as it has resulted in low steady-state levels of recombinant proteins (Demain and Vaishnav 2009; Surzycki et al. 2009; Tran et al. 2009; Sebesta and Peebles 2020; Coragliotti et al. 2011; Gregory et al. 2013; Jones and Mayfield 2013; Rasala and Mayfield 2015; Baier et al. 2018; Dyo and Purton 2018), often much less than 0.1% of the cell protein content. This drawback limits carbon partitioning toward the heterologous pathway or target protein and has a negative impact on rates and yield of product biosynthesis. Thus, true overexpression of recombinant proteins functioning in heterologous biosynthetic pathways and systems has been a barrier and a problem that we are only now beginning to overcome in this field (Formighieri and Melis 2015; Zhang et al. 2021; Hidalgo Martinez et al. 2022).

In general, there is a need to develop methods that will systematically and reliably over-express eukaryotic, including plant and human, proteins in photosynthetic microorganisms. However, the question of recombinant pathway enzymes or biopharma protein overexpression in photosynthetic microorganisms has been neglected, in spite of its central importance to synthetic biology. The problem is exacerbated because of the frequent assumption in the field that a strong promoter will automatically translate in gene overexpression (Formighieri and Melis 2014), when, in practice, SDS-PAGE and Coomassie stain measurements fail to detect presence of the transgenic protein and only sensitive Western blot analysis (Bentley et al. 2013) can offer evidence of low-level protein presence. Zhang et al. (2021) reported that, in cyanobacteria and presumably other photosynthetic microorganisms, eukaryotic plant- and animal-origin recombinant proteins are unstable and they are degraded by the host cell. The low steady-state level of such proteins, therefore, reflects the balance between the rate of synthesis and degradation, explaining why strong promoters for the expression of transgenes may be necessary but not sufficient for true protein overexpression and accumulation.

**Fig. 6:**
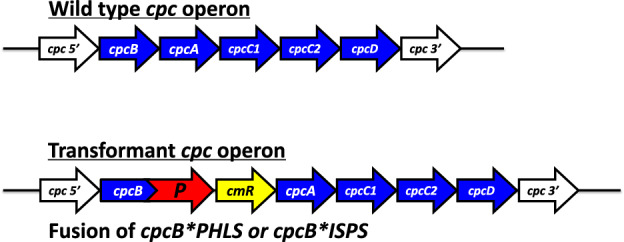
Schematic of DNA maps of the *cpc* operon in wild-type and *Synechocystis* fusion construct transformants. (Upper) The native *cpc* operon, as it occurs in wild-type *Synechocystis*, comprising the *cpcB* (phycocyanin β-subunit) and *cpcA* (phycocyanin α-subunit) DNA, as well as DNA encoding for the phycocyanin rod linker CpcC1, CpcC2, and CpcD proteins. This DNA operon configuration and sequence is referred to as the wild type (WT). (Lower). Replacement of the *cpcB* gene with fusion DNA construct cpcB*P, where P encodes for a protein of interest. The cpcB*P fusion construct is followed by DNA encoding the chloramphenicol (*cmR*) resistance cassette in an operon configuration. This transgenic DNA configuration enabled substantial accumulation of the recombinant CpcB*P fusion protein

**Fig. 7:**
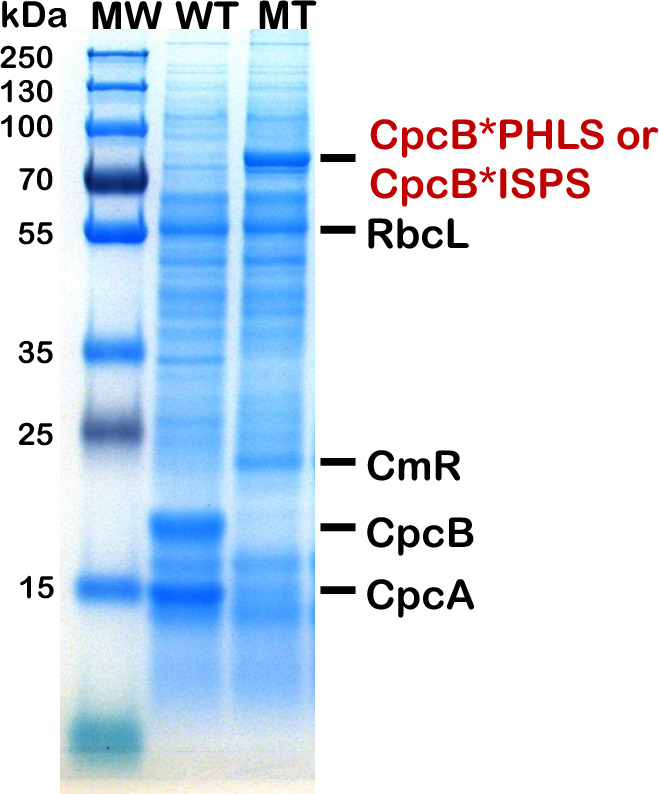
Protein expression analysis of *Synechocystis* wild-type (WT) and fusion construct transformants (MT) harboring the CpcB*PHLS or CpcB*ISPS encoding recombinant DNA. Total cellular protein extracts were resolved by SDS-PAGE and visualized by Coomassie stain. Two different versions of the fusion construct were used comprising the CpcB*PHLS or CpcB*ISPS, both of which migrate to about 83 kDa, yielding similar results. Note the equivalent amounts of the Rubisco large subunit (RbcL) in wild type and mutant, migrating to about 56 kDa, suggesting about equal loading of WT and MT proteins. Also note the presence of the CmR protein, migrating to about 23 kDa, and the absence of the CpcB protein from the ~ 19 kDa electrophoretic mobility position, as the latter is migrating to ~ 83 kDa in the MT mutants. Sample loading corresponds to 0.25 mg of chlorophyll per lane

**Fig. 8:**
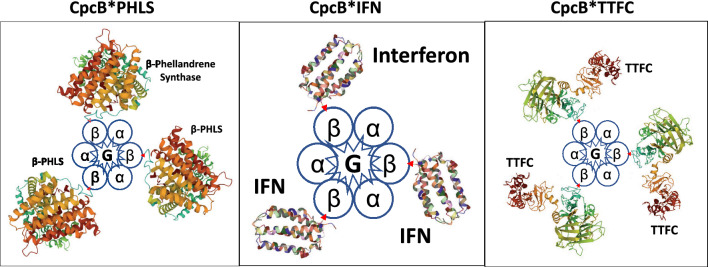
Schematic of the heterohexameric structure of recombinant protein fusions with the CpcB β-subunit of phycocyanin. Shown are the (α,β*PHLS)_3_CpcG1, (α,β*IFN)_3_CpcG1, and (α,β*TTFC)_3_CpcG1 heterohexameric complex configurations with the lavender β-phellandrene synthase, (left panel), the human interferon (middle), and the tetanus toxin fragment C (right panel). A similar configuration resulted when these heterologous proteins were fused to the CpcA α-subunit of phycocyanin. The CpcG1 linker protein (denoted by G) occupies the disk center of the respective complexes and serves to functionally link the modified phycocyanin disk to the allophycocyanin core cylinders in *Synechocystis*. Assembly of the native α,β heterohexameric complex and its functional association with the allophycocyanin core cylinders suggested that the corresponding heterologous fusion proteins localize away from the disk center and are likely placed at the periphery or emanate radially from the (α,β*PHLS)_3_, (α,β*IFN)_3_, and (α,β*TTFC)_3_ disks, thus being exposed to the medium

“*Fusion constructs as protein overexpression vectors*” proved to be unparalleled in their ability to cause substantial accumulation of recombinant proteins from plants, animals, and bacteria, as soluble and functional proteins in unicellular cyanobacteria (Formighieri and Melis 2015; 2016; Chaves et al. 2017; Betterle et al. 2020; Hidalgo Martinez et al. 2022; Hidalgo Martinez and Melis 2023). Stable recombinant protein expression reached levels in the range of 10 − 20% of the total cellular protein with this approach. Typically, the target heterologous protein “P” of interest was expressed as a fusion with the abundant CpcB β-subunit (Fig. 6) or as a fusion with the also abundant CpcA α-subunit of phycocyanin (Hidalgo Martinez and Melis 2023), which are placed in the leader sequence position. A broad range of recombinant proteins have been successfully tested, including proteins of the plant isoprenoid biosynthetic pathway, i.e., the isoprene synthase (ISPS), β-phellandrene synthase (PHLS), geranyl diphosphate synthase (GPPS), and geranyllinalool synthase (GLS), the human interferon α-2 protein (IFN), and the bacterial *Clostridium tetani* tetanus toxin fragment C (TTFC). Such fusion constructs had no adverse effect on cell fitness but they did cause rearrangement in the protein profile of phycocyanin and the phycobilisome in the transgenic cells. This is exemplified in the SDS-PAGE results of Fig. 7 where, in the wild type (WT), the Coomassie-stained dominant proteins are the CpcB 19 kDa β-subunit and CpcA 15 kDa α-subunit of phycocyanin. The latter appear to be missing from the CpcB*P fusion protein transformant (MT). Instead, the dominant protein is now the CpcB*PHLS fusion or CpcB*ISPS fusion, both migrating to about 83 kDa, clearly seen and easily quantifiable from the SDS-PAGE and Coomassie strain analysis of total protein cell extracts (Formighieri and Melis 2015; Chaves et al. 2017; Betterle et al. 2020). An initial hypothesis for such overexpression was that CpcB*P and CpcA*P fusion proteins somehow accumulate in a soluble and stable form in the cytosol of the cyanobacteria, retaining the activity of the trailing heterologous “P” protein of interest. However, work by Hidalgo Martinez and coauthors (Hidalgo Martinez et al. 2022) revealed a substantially different and previously unobvious picture, in which the CpcB*P and CpcA*P proteins assembled as functional (α,β*P)_3_ or (α*P,β)_3_ heterohexameric disks (Fig. 8), where α is the CpcA α-subunit, β is the CpcB β-subunit of phycocyanin, and *P denotes the target recombinant protein in fusion with either the native CpcB or CpcA.

The work of Hidalgo Martinez et al. (2022; 2023) further showed that the (α,β*P)_3_ and (α*P,β)_3_ heterohexameric disks are functionally attached to the *Synechocystis* allophycocyanin core cylinders, via the native CpcG1 linker protein, and efficiently absorb and transfer excitation energy from the assembled (α,β*P)_3_ or (α*P,β)_3_ heterohexameric phycocyanin subunits to the PSII reaction centers, thereby enhancing the rate of charge separation and photochemical electron transfer in the cellular thylakoid membrane. This discovery demonstrated that cyanobacterial cells can tolerate heterologous recombinant proteins, when the latter are in a fusion construct configuration with essential cellular proteins, e.g., phycocyanin. The latter, in this case, is needed by the cell for light absorption and excitation energy transfer to a photosystem, thus enabling the substantial and stable accumulation of the associated transgenic proteins (Hidalgo Martinez and Melis 2023).

### Outlook

In recent years, the field of cyanobacterial biotechnology has emerged as a transformative process, receiving considerable attention due to its potential to revolutionize synthetic biology with the generation of sustainable and renewable products. Cyanobacteria are adept at dual roles as photocatalysts and cellular processors, exhibit the remarkable ability to both photosynthesize, and convert inorganic carbon into an array of valuable bio-products. An aspect that sets this technology apart is its carbon-negative footprint, making it a tool in the mitigation of climate change. Moreover, its versatility and easiness to use allows for cost-effective product generation, with applicability spanning across the globe, offering a distributed source of employment and economic growth, particularly benefiting developing countries.

Cyanobacteria cell factories hold promise in several key areas with far-reaching societal impacts. These include the synthesis of chemical compounds essential for the flavor and fragrance industries, as well as providing feedstock for the synthetic chemistry industry. Furthermore, their potential extends to the production of specialty enzymes and proteins, catering to the biopharmaceutical sector’s diverse needs, encompassing diagnostics, antigens, antibodies, growth factors, and more. Additionally, cyanobacteria offer a sustainable alternative to conventional plastics through the production of thermoplastics, like the biodegradable polyhydroxybutyrate (PHB), thus addressing the global plastic pollution crisis.

However, to fully harness the potential of cyanobacterial cell factories, significant research efforts are required. Overcoming challenges such as enhancing the simultaneous overexpression of multiple heterologous enzymes to optimize the redirection of endogenous substrates toward desired products is paramount. Moreover, increasing the yield of specialty enzymes and recombinant biopharmaceutical proteins to levels exceeding the current 10–15% of the total cellular protein content is a critical milestone. Achieving these advances will facilitate the seamless transition of cyanobacterial biotechnology from the research laboratory to the private sector, unlocking a sustainable future for both industry and the environment.

## Data Availability

All data and material are available from the corresponding author.

## Abbreviations

AP: Allophycocyanin
PC: Phycocyanin
*cpcA*: Gene encoding the PC α-subunit
*cpcB*: Gene encoding the PC β-subunit
*cpcG1*: Gene encoding the proximal PC-AP linker protein;
*Synechocystis*: *Synechocystis* sp. PCC 6803
PBS: Phycobilisome
PSII: Photosystem II
Chl: Chlorophyll
WT: Wild type
